# Designing a regenerable stimuli-responsive grafted polymer-clay sorbent for filtration of water pollutants

**DOI:** 10.1080/14686996.2018.1499381

**Published:** 2018-08-16

**Authors:** Ido Gardi, Yael G. Mishael

**Affiliations:** Department of Soil and Water Science, The Robert H. Smith Faculty of Agriculture, Food and Environment, Hebrew University of Jerusalem, Rehovot, Israel

**Keywords:** Grafted polymer clay composites, pollutant adsorption, filtration, regenerable sorbents, 20 Organic and soft materials (colloids, liquid crystals, gel, polymers), 103 Composites, Pollutants adsorption, Water treatment, 301 Chemical syntheses / processing

## Abstract

A novel, stimuli-responsive composite, based on poly(4-vinylpyridine) (PVP) brushes, end-grafted to montmorillonite clay (GPC), was designed as a regenerable sorbent for efficient removal of pollutants from water. We characterized the novel composite sorbent and its response to pH, employing Fourier transform infrared, X-ray photoelectron spectroscopy, X-ray diffraction, thermogravimetry analysis and zeta potential measurements. In comparison with conventional, electrostatically adsorbed PVP composites (APC), the GPC presented superior characteristics: higher polymer loading without polymer release, higher zeta potential and lower pH/charge dependency. These superior characteristics explained the significantly higher removal of organic and inorganic anionic pollutants by this composite, in comparison with the removal by APC and by many reported sorbents. For example, the filtration (20 pore volumes) of selenate by GPC, APC and a commercial resin column was complete (100%), negligible (0%) and reached 90% removal, respectively. At low–moderate pH, the grafted polymer undergoes protonation, promoting pollutant adsorption, whereas at high pH, the polymer deprotonates, promoting pollutant desorption. Indeed, ‘in-column’ regeneration of the GPC sorbents was achieved by increasing pH, and upon a second filtration cycle, no reduction in filter capacity was observed. These findings suggest the possible applicability of this stimuli-responsive sorbent for water treatment.

## GRAPHICAL ABSTRACT

**Figure:**
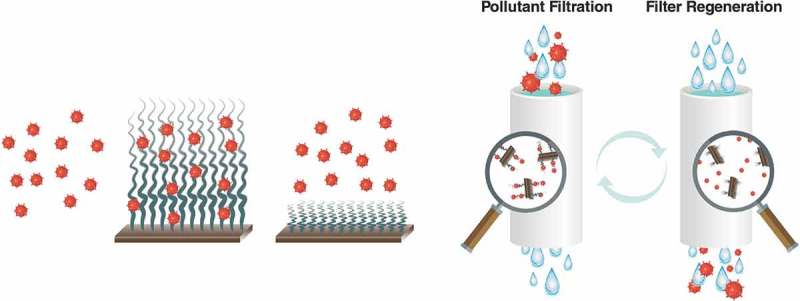

## 1. Introduction

One of the greatest environmental challenges of the twenty-first century is meeting the growing demand for high-quality water. Therefore, many resources are directed towards improving and developing water treatment technologies [1,2]. A large number of studies have aimed to develop new sorbents for pollutant removal [3–6], such as organically modified clay minerals, termed organoclays [7,8]. More recently, adsorbed polymer clay composites have often exceeded the performance of commercial sorbents and have therefore drawn attention as sorbents for water treatment [9–11]. We have demonstrated that composites based on protonated poly(4-vinylpyridine) (PVP) electrostatically adsorbed, by cation exchange, to montmorillonite clay (MMT) perform better than other polymer clay composites [12] due to the range of interactions that PVP can form with micropollutants. In addition, the pyridine group of the polymer can be further functionalized to improve composite performance [13,14]. Indeed, adsorbed functionalized PVP–MMT composites were efficient sorbents for filtration of a variety of pollutants including dissolved organic matter [14], pyrene [15], phenolic derivatives [16], pharmaceuticals [13] and pesticides [12,17].

Hereby, a novel approach is proposed for the design of regenerable polymer-MMT composite sorbents, based on covalently grafting a stimuli-responsive polymer to clay. Grafting of functional polymers to the clay mineral surfaces improves the ability to tune the properties of clay mineral surfaces [18]. Such anchored polymers form a stationary phase which may enhance the performance of the composite [19]. Numerous polymer-grafted nanoparticles, such as graphene [20], gold [21,22], magnetite [23,24], metal oxides [25,26] and natural clays [27–29], have been reported and used for a wide range of applications.

An additional advantage of grafting is that higher loadings can be achieved, compared with adsorbing polymers. Furthermore, polymers may respond to external stimuli, for instance pH [30,31], temperature [32], ionic strength [33] or infrared light [21]. Covalently grafting such polymers to a surface will result in stable stimuli-responsive polymer brushes [34,35] that respond to a stimulus by changing surface chemistry and structure and therefore may adsorb or desorb pollutants. Indeed, the sorption properties of grafted polymer brushes on clays have been recently acknowledged [28,36,37]. However, none of these studies demonstrated the application of these sorbents in filtration columns, which is the most probable mode in which any candidate sorbent will be applied and compared with other sorbents [34]. Furthermore, in many cases, the limiting factor in applying such sorbents is their inefficient reuse [38,39] since the regeneration should selectively remove the pollutant while the polymer must remain intact. Hence, this challenge has yet to be properly resolved [38].

In this study, we developed a regenerable, pH-responsive sorbent based on PVP brushes grafted to montmorillonite (GPC). The characteristics of the new GPC were compared to those of conventional, electrostatically adsorbed PVP clay composites (adsorbed PVP composite [APC]). The GPC was designed to respond to pH; at low–moderate pH, the polymer undergoes protonation, promoting pollutant adsorption, whereas at high pH, the polymer deprotonates, promoting pollutant desorption. The removal potential of six water micropollutants, selenate, arsenate, sulfentrazone, atrazine, methyl blue and eosin-Y by GPC, was explored. The filtration of oxyanions (selenate) and organic anions (eosin-Y) by GPC columns was more efficient than by APC columns or by commercial sorbent columns. Furthermore, ‘in-column’ regeneration of GPC, induced by pH, was successfully demonstrated.

## 2. Experimental section

### 2.1. Materials

Montmorillonite clay, SWy-2 (MMT), was purchased from Source Clay Repository (Clay Minerals Society, Columbia, MO, USA). 3-Aminopropyltrietho-xysilane (ATPES), 2-bromoisobutyryl-bromide (BIB), 4-vinylpyridine (4-VP), PVP, ethanol, glycerol, anhydrous toluene and anhydrous trimethylamine (TMA) (high-performance liquid chromatography grade), potassium arsenate (KH_2_AsO_4_), eosin-Y (C_20_H_6_Br_4_Na_2_O_5_), sulfuric acid (98%) and copper salts (CuCl H_2_O and CuCl_2_) were purchased from Sigma Aldrich Ltd., Rehovot, Israel. Tris(2-dimethylaminoethyl) amine, potassium selenate (K_2_SeO_4_) and methyl blue (C_37_H_27_N_3_Na_2_O_9_S_3_) were supplied by Alfa Aesar, Yehud, Israel. Atrazine (C_8_H_14_ClN_5_) 98% and sulfentrazone (C_11_H_10_Cl_2_F_2_N_4_O_3_S) 91.3% were obtained from Makhteshim-Agan Industries Ltd., Tel Aviv, Israel. Granular activated carbon (GAC) Hydraffin 30 N Donau Carbon was obtained from Reactive Ltd., Shahak Industrial Park, Israel. Drinking water grade anion exchange resin (AMBERLITE™ PWA15) produced by Dow Water & Process Solutions was received from TREITEL chemical engineering Ltd., Tel Aviv, Israel..

### 2.2. Preparation of adsorbed PVP composite

The preparation of APC was previously reported [12]. Briefly, PVP (3 g) was dissolved in 14 mM H_2_SO_4_ solution. The polymer solution was then slowly added to MMT suspension (1.67 g/L). The solid was separated from the supernatant by centrifugation, washed and freeze-dried.

### 2.3. Preparation of grafted PVP composite

**Figure 1.**
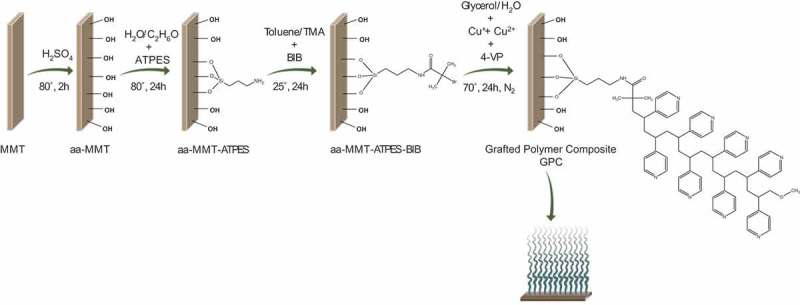
Schematic of the preparation of the grafted PVP montmorillonite composite (GPC).

The stages in montmorillonite surface modification are illustrated in Figure 1. *Acid activation and amination of montmorillonite surface*: Oven dry MMT (15 g) was suspended in 300 mL water at 80 °C. Sulfuric acid (3 mL; 98%) was added to the suspension under stirring (120 min). The acid-activated montmorillonite (aa-MMT) was then washed with distilled water, freeze-dried and stored in a desiccator. Thereafter, dry aa-MMT (10 g) was suspended in a 500 mL mixture of ethanol and water (3:1, volume ratio) under reflux at 80 °C. ATPES (6.24 mL) was mixed in a 100 mL ethanol/water mixture and added dropwise, and the temperature was raised to 95 °C for 24 h. The solids were separated by centrifugation, washed thoroughly with ethanol and water and freeze-dried. Dry ATPES grafted aa-MMT (aa-MMT–ATPES) was stored in a desiccator.

*Initiation of aminated montmorillonite*: aa-MMT–ATPES (5 g) was suspended in 40 mL anhydrous toluene and 10 mL anhydrous TMA. The suspension was cooled down to 0 °C in an ice bath. BIB (5 mL) mixed in 30 mL anhydrous toluene was added dropwise to the cold suspension. Next, the ice bath was removed, and the reaction was carried out at ambient temperature for 24 h. aa-MMT–ATPES–BIB was then separated by centrifugation, thoroughly washed with toluene and acetone, dried at 105 °C overnight and kept in a desiccator.

*Surface initiated – atom transfer radical polymerization (SI-ATRP) of PVP from initiated montmorillonite*: aa-MMT–ATPES–BIB (1.1 g) was suspended in a 140 mL mixture of glycerol and water (1:1, volume ratio). CuCl H_2_O (20.4 mg), CuCl_2_ (59.4 mg) and Tris(2-dimethylaminoethyl) amine (384 µL) were added to the suspension. The suspension was degassed by three cycles of freeze-pump-thawing, and the frozen suspension thawed under a N_2_ stream. Monomers of 4-VP (3.2 mL) were mixed in a 100 mL glycerol–water mixture (1:1, volume ratio), degassed as above and the monomer solution was added dropwise to the initiated clay suspension. The polymerization reaction was carried out at 90 °C under N_2_ atmosphere for 24 h. The polymerization reaction was terminated by dropwise addition of a 1:9 HCl and methanol mixture (5 mL). Ethanol (100 mL) was added to the suspension and stirred for 2 h, and the solids were separated using centrifugation. The grafted PVP-clay composite (GPC) was thoroughly washed with ethanol and an ethanol/water mixture containing 1% HCl, freeze-dried and stored in a desiccator.

### 2.4. Characterization

Composite characterization, at the different preparation stages, was performed as follows: Fourier transform infrared (FTIR) spectra were recorded using a Nicolet 6700 (Thermo Waltham, MA, USA) FTIR spectrometer equipped with an attenuated total reflection module. The surface zeta potential as a function of pH was measured using a Malvern, UK, Zetasizer Nano ZS combined with an auto titrator. The pH of the composite suspensions (1 g L^−1^) was adjusted to 2.5 with HCl and titrated with 0.01 M NaOH to pH 11. Elemental analysis of C and N was performed with a CHNSO analyser (FlashEA 1112, Thermo, Waltham, MA, USA). X-ray photoelectron spectroscopy (XPS) survey and high-resolution measurements around N, C and Br binding energies were performed with a Kratos Analytical (Manchester, UK) Axis Ultra (XPS/ESCA). X-ray diffraction (XRD) patterns of oriented samples were recorded with an X-ray diffractometer (Philips, Almelo, Netherlands, PW1830/3710/3020). Thermal gravimetric analysis (TGA) at 60–900 °C was carried out with a Q500 TGA (TA Instruments, New Castle, USA) in high-resolution mode (HR sensitivity 1.5, ramp of 35 °C min^−1^, res 3.5) under N_2_ atmosphere. The thermal gravimetric derivative (DTG) was calculated using TA QSeries Advantage software, Universal Analysis package. Photographs of composite suspensions at different pH values were taken after suspending 1 g L^−1^ of each composite in 0.04 M of HCl, acetate buffer or NaOH, which resulted in suspension pH values of 3, 5.8 and 10, respectively.

### 2.5. Filtration

The filtration of selenate or eosin-Y was performed in glass column (20 cm in length and 1.6 or 1 cm in diameter, respectively) duplicates. The columns were packed with 1:100 sorbent (GPC, APC or commercial): fine quartz sand (50–150 mesh). Each column was slowly saturated from below using acidified water (HCl 0.01 M).

#### 2.5.1. Filtration

Selenic acid (0.2 or 0.165 mM) and eosin-Y (0.04 or 0.1 mM) were pumped through the columns at a flow rate of 2.5 or 1.5 cm min^−1^, and effluent samples were collected over time and analysed for pollutant concentration.

#### 2.5.2. Desorption

Desorption of the pollutants from the columns was performed by pumping NaOH 0.01 M solution (pH 11.6) at the same flow rate. Effluent samples were collected over time and analysed for pollutant concentration and pH. Desorption was carried on until the effluent pH was equal to the feed pH.

Reactivation was performed using HCl 0.01 M solution (pH 2.8) at the same flow rate. Samples were collected over time and analysed for pollutant concentration. Activation ceased when the effluent pH was equal to the feed pH. Then, fresh pollutant solution was filtrated as described in the previous section.

### 2.6. Adsorption isotherms

Potassium selenate, potassium arsenate, methyl blue, eosin-Y, atrazine and sulfentrazone were each added at increasing concentrations (0.001–0.86 mM) to 2 g L^−1^ GPC, in triplicates. The samples were agitated for 24 h and centrifuged, and the supernatant was analysed.

Desorption of each pollutant from the GPC was determined as follows: 0.01 mmol pollutant per 1 g composite was added to the composite suspension. After 24 h, the samples were centrifuged, and the supernatant was separated and analysed. NaOH (0.01 M) was added to the sedimented solids, and the samples were agitated. After 2 h, the samples were centrifuged, and the supernatant was analysed. Langmuir adsorption model was fitted (least squares fitting method) to the adsorption isotherms results to extract the adsorption coefficient K_L_ (M^−1^) and maximal capacity (mmol kg^−1^) for each pollutant.

### 2.7. Pollutant analysis

Oxyanions (selenate and arsenate) were detected with the axial inductively coupled plasma optical emission spectrometer ARCOS (Spectro GmbH, Kleve, Germany) and analysed using ‘Smart Analyzer’ (4.02.0831) software. The organic dyes were quantified with a UV–Vis spectrophotometer, adjusting solution pH by adding 1 mL of potassium phosphate buffer (pH 7) to 1 mL sample, methyl blue (*λ* = 595 nm) and eosin-Y (*λ* = 340 nm). Atrazine and sulfentrazone were analysed by Agilent 1200 series high-performance liquid chromatography setup equipped with a C_18_ column and a diode array detector (*λ*_atrazine_ = 222 nm and *λ*_sulfentrazone_ = 256 nm). The mobile phase consisted of 70:30 acetonitrile and water for atrazine detection and 60:40 acetonitrile and water +0.1% H_2_PO_4_ for sulfentrazone detection.

## 3. Results and discussion

### 3.1. Preparation and characterization of GPC

**Figure 2.**
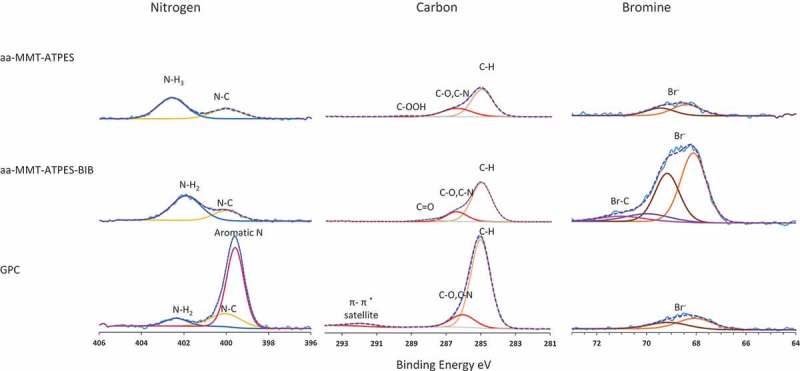
High-resolution XPS measurements of N, C and Br binding energies in aa-MMT–ATPES, aa-MMT–ATPES–BIB and GPC (top to bottom).

**Figure 3.**
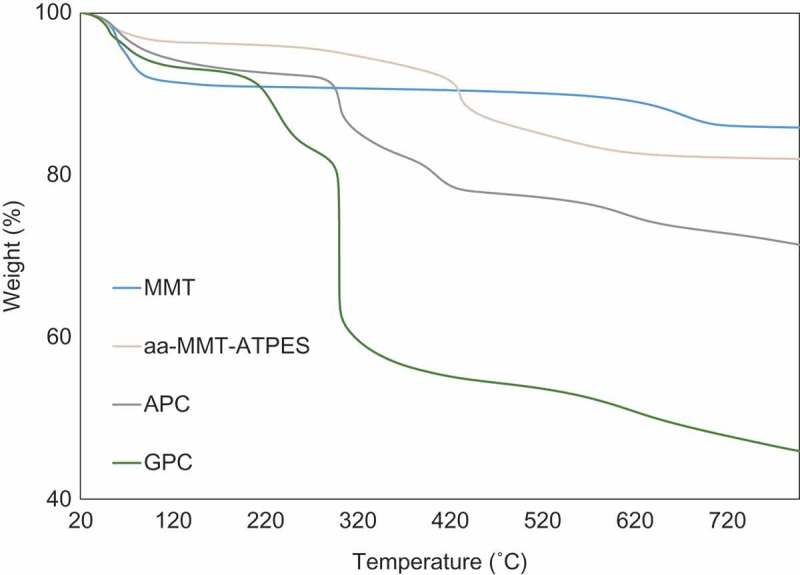
Thermal gravimetric analysis of MMT, aa-MMT–ATPES, APC and GPC.

The preparation of GPC is presented in Figure 1 and involved acid activating of montmorillonite (aa-MMT), grafting ATPES (aa-MMT-ATPES), surface initiating with BIB (aa-MMT-ATPES-BIB) and, finally, vinylpyridine polymerizing (SI-ATRP) [40] resulting in polymer-grafted composite (GPC). The different stages of the synthesis were characterized by zeta potential measurements (Figure S1A), X-XRD (Figure S1B), FTIR (Figure S2), elemental analysis (Table S1), XPS (Figure 2) and TGA (Figure 3). Based on these measurements, the synthesis stages were illustrated in Figure 1.

Here, we describe the four synthesis stages systematically followed by measuring the distribution of nitrogen, carbon and bromine binding energies by high-resolution XPS (Figure 2). The nitrogen, carbon and bromine content on MMT and aa-MMT is negligible and therefore not presented. The nitrogen binding energies on the surface of aa-MMT–ATPES were recorded at 400 and 403 eV with a 1:3 peak ratio, assigned to N-C and N-H (of NH_3_ functional group) bonds attributed to the presence of ATPES (primary amine) on the surface. Carbon binding energies recorded at 285 and 287 eV confirmed that C-H, C-O or C-N bonds were the main carbon bonds on the surface. The low concentrations of bromine at the sample surface (i.e. lower than 0.23 mol percentage) are attributed to impurities.

Initiation of aa-MMT–ATPES with BIB (aa-MMT–ATPES–BIB) is reflected in a small peak attributed to C=O of carbon binding energies and in a shift of the peak assigned to NH_3_ (403 eV), to a binding energy assigned to NH_2_ (402 eV), supporting BIB binding to NH_3_. Furthermore, the 5/2 peak at 68 eV that appeared in all the samples indicated the presence of small amounts of Br**^−^** bound to the surface. An additional Br bond that can be assigned to C-Br was recorded at the surface of aa-MMT–ATPES–BIB, as indicated by the 5/2 peak at 70 eV [41,42].

This bond (C-Br) was not detected after vinylpyridine polymerization, supporting the reaction course. An increase in aromatic N at 399 eV, constituting nearly 90% of the N bonds in GPC, is attributed to pyridine monomers anchored to the surface. The presence of pyridine is also reflected by the appearance of an indicative peak at 292 eV corresponding to C-π* satellite electrons. Polymerization-induced clay exfoliation (suggested by XRD measurements, Figure S1B) as reported for poly(ethyl acrylate) brushes grafted to montmorillonite [43].

The loading of the grafted polymer was obtained by thermal gravimetric analysis of bare montmorillo-nite (MMT), (aa-MMT-ATPES), GPC and APC (Figure 3(a)). The first derivative of the thermograms (DTG) for GPC from an acidic pH and from a basic pH emphasizes similarly high polymer loading (no polymer release observed); the sharp peaks indicate a homogeneous brush phase (Figure S3). Grafting ATPES resulted in mass loss of 8% at a temperature range of 350–500 °C, well above the boiling point of ATPES (217 °C) and distinguished from MMT dehydroxylation (600–700 °C) [44]. The increase in ATPES thermal stability is due to covalent grafting and perhaps also to intercalation between clay platelets (Figure S1B). The weight loss around 200 °C may be attributed to BIB residues. The GPC demonstrated thermal stability of the grafted polymer, with weight loss at a temperature range of 250–500 °C, in comparison with the non-grafted monomer and polymer which have boiling points of 63.5 °C and 260 °C, respectively. The substantial additional mass loss obtained for GPC (at 250–500 °C) is attributed to the high loading of PVP brushes, 31% (w/w), which is within the range of previous reports on vinylpyridine grafting to halloysite [45]. The loading of the grafted polymer in GPC is approximately two times higher than the loading of the adsorbed polymer in APC (15%).

### 3.2. Effect of pH on GPC and APC

**Figure 4.**
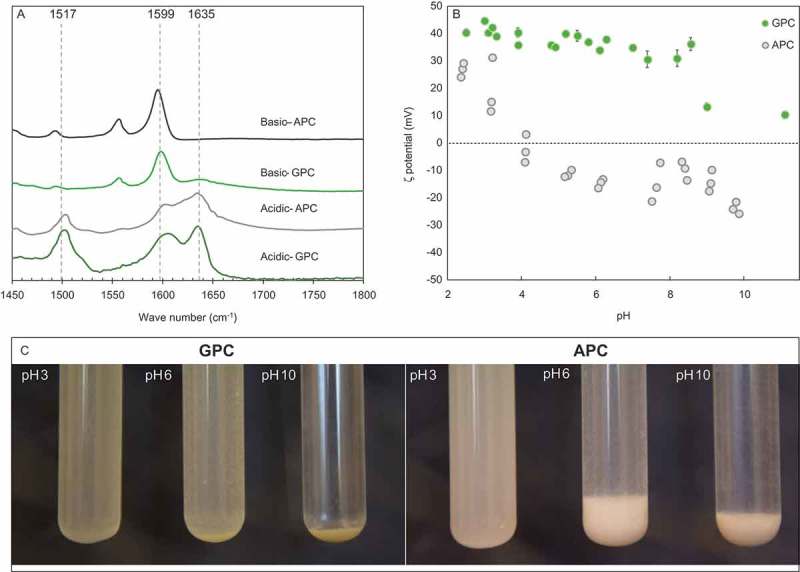
(a) FTIR spectra of APC and GPC at pH 3 and 11. (b) GPC and APC zeta potential as function of suspension pH. (c) Photographs of GPC and APC suspensions at pH 3, 6 and 10.

Protonation and deprotonation of PVP in both clay composites (APC and GPC) at pH 3 and pH 11, respectively, are confirmed by FTIR measurements. In acidic conditions, a sharp peak at 1635 cm^−1^ assigned to the pyridinium N=C bond appears in both composites (Figure 4(a)) [46]. The characteristic pyridine peak at 1599 cm^−1^ was shifted to a higher wave number, 1610 cm^−1^ which may be assigned to pyridine associated (hydrogen bonds) with water [47,48]. The broader peak of associated pyridine in APC spectra may be a result of a larger fraction of monomers interacting with the clay surface. At basic conditions, the 1635 cm^−1^ band diminishes and the characteristic pyridine N=C band at 1599 cm^−1^ appears.

The zeta potential of APC and GPC at pH 2 reached 30 and ~40 mV, respectively; the higher charge of GPC is attributed to a higher polymer loading (Figure 4(b)). The zeta potential of APC gradually decreases with an increase in suspension pH. At pH 4, charge reversal is reached, and at pH 8, the zeta potential is approximately −20 mV. The high positive zeta potential of GPC remained constant over a wide range of pH values. At pH 8, GPC undergoes a step-like reduction in zeta potential, reaching a value of +10 mV.

The p*K*_a_ of PVP in solution is 3.0 ± 0.9 and depends on chain length and solution chemistry [49,50]. A similar isoelectric point is observed for the adsorbed PVP (APC). However, the deprotonation of grafted PVP (GPC) is reached at a significantly higher pH. The grafted PVP phase contains a lower pH than the bulk solution, as previously described in the case of polymer brushes [51], and therefore the positive zeta potential of GPC remains at solution pH values higher than the polymer p*K*_a_ (Figure 4(b)).

The difference in zeta potential response to pH of the two PVP–MMT composites, adsorbed (APC) or grafted (GPC), can be observed by the naked eye (Figure 4(c)). Both APC and GPC suspensions are highly stable at pH 3, due to the strong electrostatic repulsion between the particles. Meanwhile, the particles (APC and GPC) aggregate and precipitate at pH 10 due to the reduction in particle charge. At pH 6 however, the PVP adsorbed in APC deprotonates and the particles rapidly precipitate, whereas the grafted PVP remain protonated and the GPC suspension is stable.

The above observations suggest that filtration of anionic pollutants at ambient pH will be significantly higher by the GPC sorbent in comparison to filtration by the APC sorbent, not only due to the higher loading in the GPC sorbent but also due to the high protonation of GPC up to pH 8.

### 3.3. Pollutant filtration by APC and GPC composite columns

**Figure 5.**
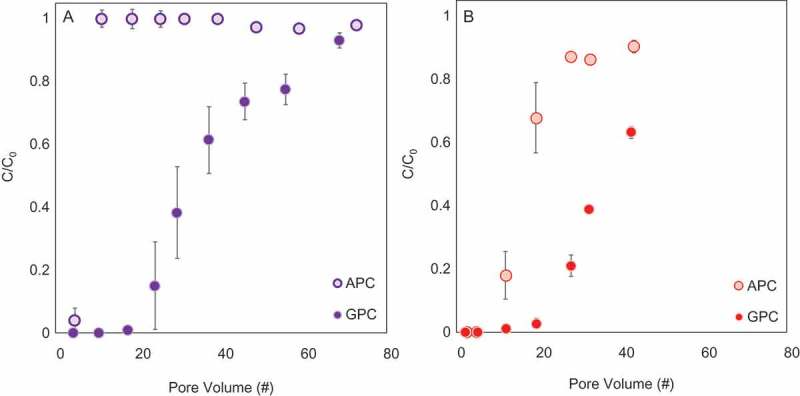
Filtration of (a) selenate (0.2 mM) or (b) eosin-Y (0.1 mM) through columns of APC or GPC (mixed with sand) presented as emerging pollutant concentrations (C/Co) as a function of the number of column pore volumes.

The removal of the oxyanion, selenate and the anionic dye eosin-Y by filtration through columns containing APC or GPC was measured (Figure 5). The pH of the effluent in all cases was monitored and found to similarly increase from an initial pH of 3.5 to the pH value of the feed solution.

Selenate and eosin-Y filtration was more efficient by the GPC columns in comparison to the APC columns. For example, the removal of both selenate and eosin-Y, after the filtration of 20 pore volumes, by GPC column was complete (100%), while the removal of selenate by the APC column was negligible (0%) and of eosin-Y reached 30%. The rapid deprotonation of adsorbed PVP (APC) diminishes the electrostatic attraction to selenate, and instantaneous breakthrough from the APC columns is obtained. For eosin-Y, other interactions (such as π–π interactions and Van der Waals interactions) can also contribute to the adsorption, and therefore, a more moderate breakthrough from the APC columns is observed. The superior performance of GPC columns is attributed to this composite’s high positive zeta potential even at neutral/environmental pH (Figure 4(b)) and higher capacity per gram sorbent. Therefore, the potential of the GPC as regenerable sorbents was further investigated.

### 3.4. Stimuli response of GPC – pollutant adsorption and desorption

**Figure 6.**
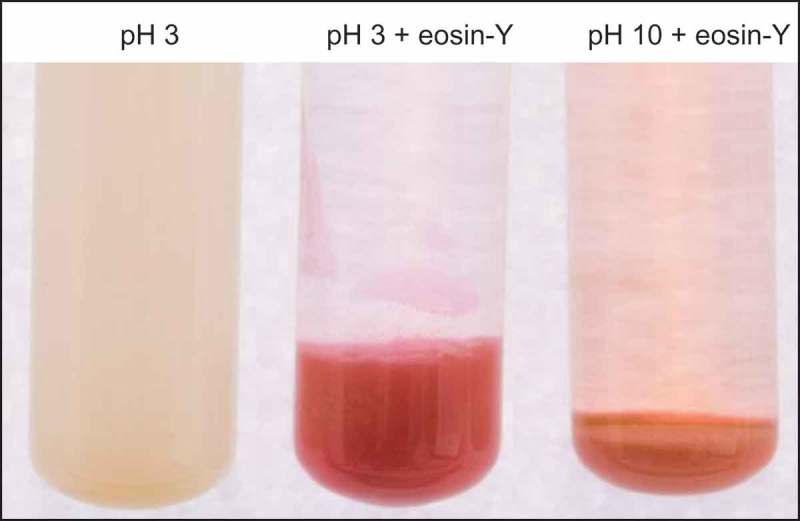
GPC suspension at pH 3, GPC (after centrifugation) with eosin-Y adsorbed at pH 3 and desorbed at pH 10.

As described above (Figure 4(c)), the grafted polymer protonates and deprotonates at low–moderate pH and high pH, respectively. The change in pH most likely results also in a polymer conformational change similar to polymer brushes in a bad solvent [52]. At low–moderate pH, the polymer brushes repel each other, promoting their extension into solution and increasing particle volume. Meanwhile, at high pH, the chains collapse and decrease the particle volume (Figure 6). The slightly higher onset temperature of polymer decomposition (obtained from TGA measurements), for the GPC from a basic pH, in comparison to the acidic GPC, supports a more condense polymer structure at a basic pH (Figure S3).

These GPC surface properties, i.e. charge and conformational changes, can trigger anionic pollutant adsorption at low–moderate pH and desorption at high pH. For example, the adsorption and desorption of the anionic dye eosin-Y at low and high pH, respectively, are visible in Figure 6.

### 3.5. Pollutant filtration by GPC and column regeneration

**Figure 7.**
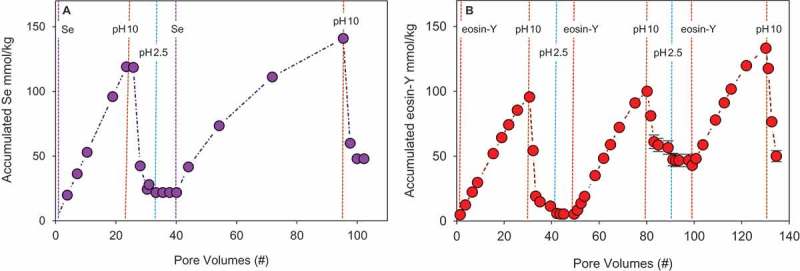
The accumulated amount of (a) selenate (0.16 mM) and (b) eosin-Y (0.04 mM) on GPC as a function of the number of pore volumes filtrated through the column. Dashed lines indicate a change in the feed solution pumped through the columns.

The concept of pH-triggered pollutant adsorption and desorption to/from GPC was also demonstrated by filtration of water contaminated with selenate or eosin-Y followed by in-column regeneration and reuse. Pollutant removal and pollutant desorption (by passing a basic solution) followed by column activation (by passing an acidic solution) and a second cycle of pollutant removal are presented as a function of number of pore volumes passed through the columns (Figure 7). The filtration of selenate and eosin-Y by the GPC columns was compared to the filtration by the recommended commercial sorbents – GAC (Hydraffin 30 N Donau Carbon) and resin (AMBERLITE™ PWA15) – for these two pollutants, respectively (Table S3). The filtration of both selenate and eosin-Y by GPC was extremely high (100%) and displayed high adsorption capacity and affinity, while the removal by the commercial sorbents reached only 90% and 5% for selenate and eosin-Y, respectively (Table S3). Furthermore, although the pH of the effluent increased (pH 4–6) during pollutant filtration, the removal was not reduced because of the positive zeta potential at this pH range (Figure 4(b)). The pollutant desorption facilitated column regeneration and reuse.

The regeneration of the GPC columns was achieved with an abrupt release of both pollutants. The eluting concentrations of eosin-Y and selenate were 16 and 7 times higher than the initial pollutant concentrations, respectively. The efficient desorption required only a small volume of water, reaching 80% and 90% of the total desorbed amount within 4 pore volumes. Furthermore, the first-regeneration cycles desorbed 90% and 95% of adsorbed selenate and eosin-Y, respectively, and although the second cycle of regeneration yielded less desorption, the same filter capacity was reached. Thus, pollutant removal, ‘in-column regeneration’ and sorbent reuse were repeatedly achieved. In the case of selenate, 130 mmol kg^−1^ (that is, 100%) was removed from approximately 0.5 L. In the case of eosin-Y, 88% was removed during the first and second filtration cycles; however, slightly lower removal was obtained in the third cycle (81%) due to insufficient desorption.

### 3.6. Adsorption and desorption of pollutants

To evaluate the wide-scale applicability of GPC as a regenerable sorbent, the adsorption and desorption of an array of pollutants – arsenate, sulfentrazone, atrazine and methyl blue – were measured in addition to that of selenate and eosin-Y (Table 1). The results were fitted to the Langmuir adsorption model (Figure S4, Table 1). The Langmuir model could not converge to fit methyl blue adsorption isotherm due to its nearly complete adsorption to GPC at the concentrations tested; therefore, the maximal capacity of GPC in this case was estimated as the highest measured adsorbed amount (Table S2).

**Table 1.** Pollutant chemical properties at pH 3 and 10 [53], as well as the percent of adsorbed pollutant to GPC (0.01 mmol pollutant; 1 g GPC) at pH 3 and the amount desorbed at pH 10.

	Pollutant	Arsenate	Selenate	Methyl blue	Eosin-Y	Atrazine	Sulfentrazone
	Ring count	0	0	6	4	1	2
pH 3	H – acceptors sites	4	5	11	3	5	4
	Charge^a^	−0.5	−2	−2	−0.4	0.5	0
	K_L_ (mM^−1^)^b^	2.7	2.6	NA	259.4	7.4	44.8
	Q_max_ (mmol kg^−1^)^b^	230	1232	>196.3*^c^*	123	17	18
	*R*^2b^	0.99	0.99	NA	0.94	0.97	0.94
	Adsorbed (%)	53 ± 1	92 ± 1	98 ± 1	77 ± 3	10 ± 5	11 ± 6
pH 10	H – donor sites	0	0	3	0	2	1
	Charge^a^	−2	−2	−3	−2	0	−1
	Desorbed (%)	94 ± 1	89 ± 2	40 ± 5	92 ± 4	38 ± 19	70 ± 3

^a^Average molecular charge at solution pH.

^b^Langmuir adsorption model fitting results.

^c^Measured amount

The adsorption coefficient (K_L_) of the composite was higher in the case of the organic molecules compared with the inorganic molecules, as previously reported for the adsorption of phenol and phosphate into organo-bentonite [54].

The adsorption capacity (Q_max_) of the non-ionic organic pollutants, atrazine and sulfentrazone, was similarly low (up to 18 mmol kg^−1^), indicating that hydrogen bonds [12], π–π interactions and Van der Waals interactions did not induce efficient adsorption. The higher adsorption capacity of the anionic pollutants, organic and inorganic, indicates that the main driving force is electrostatic.

The adsorption of the oxyanions reached 150–300 mmol kg^−1^, higher than reported for many other sorbents [55–61] (Table S2). The adsorption of selenate (93%) is superior to that of arsenate (53%) due to the former’s higher negative charge at pH 3 (−2 vs. −0.5). Likewise, the adsorption of methyl blue (98%) was generally higher than that of eosin-Y (77%) (charges at pH 3 were −2 vs. −0.4, respectively). The organic pollutants may also form π–π and Van der Waals interactions, explaining the nearly complete adsorption of methyl blue, in comparison to selenate, although they had the same charge. For the same reasons, the adsorption of eosin-Y is higher than that of arsenate.

The interactions at pH 3 that induce adsorption, in their absence at pH 10, desorption is induced. Upon pyridinium de-protonation, the positive charge is reduced, and pyridine becomes an H-bond acceptor. The affinity of arsenate and selenate to GPC is radically diminished at pH 10 as electrostatic attraction is reduced, resulting in efficient desorption. The desorption of eosin-Y was high as well, due to reduction in electrostatic attraction but also due to an increase in its solubility at pH 10. The reduction in electrostatic attraction along with the increase in solubility of methyl blue should promote its desorption as well; however, less desorption was obtained since methyl blue is an H donor at pH 10, while PVP is an H acceptor. Similarly, atrazine and sulfentrazone exhibited low desorption due to the ability to form hydrogen bonds, as their N-H sites as H-donors to PVP. These results (Table 1) suggest that the main interactions governing adsorption to GPC are electrostatic attraction and, in their absence, desorption is triggered.

## 4. Conclusions

We designed a novel, grafted PVP–MMT composite (GPC) by SI-ATRP. In comparison with conventional, electrostatically adsorbed PVP–MMT composites (APC), the grafted composite presented superior characteristics: higher polymer loading without polymer release, higher zeta potential and lower pH/charge dependency. These superior characteristics can explain the significantly higher removal of organic and inorganic anionic pollutants, by GPC. The sharp changes in surface properties upon pH stimulus triggered pollutant adsorption at low–moderate pH and desorption at high pH. Furthermore, the filtration of selenate and of eosin-Y by the GPC composite columns was significantly more efficient than by APC or commercial sorbent columns and was not compromised after the rapid ‘in-column’ regeneration at high pH. Finally, this study suggests that the employment of stimuli-responsive composite sorbents in filtration columns is a promising and underexplored opportunity for sustainable water treatment. Further research should explore the simultaneous removal of many pollutants from contaminated water, optimize the in-column filtration and regeneration and evaluate upscaling. The results presented here demonstrate a novel approach for enhanced performance and functionality of composites which could be adopted for the design of many other clay hybrid materials.
