# Supplementary material for: Designing a regenerable stimuli-responsive grafted polymer-clay sorbent for filtration of water pollutants
Source: Sci Technol Adv Mater. 2018 Aug 16;19(1):588–98. doi: 10.1080/14686996.2018.1499381 (PMC6104616; doi:10.1080/14686996.2018.1499381)
Supplement: Supplemental Material [file TSTA_A_1499381_SM3459.pdf]

Regenerable stimuli responsive grafted polymer-clay sorbent for filtration of water pollutants

Ido Gardi, Yael G. Mishael*

Department of Soil and Water Science, The Robert H. Smith Faculty of Agriculture, Food and Environment, Hebrew University of Jerusalem, Rehovot 76100, Israel.

Supporting information

Table of Contents

1. Preparation and characterization of poly(4-vinylpyridine) brushes grafted to montmorillonite........ 1

GPC Adsorption Performances in Comparison with Other Sorbents........ 6

Adsorption Capacity at Equilibrium........ 6

Filtration Performances, Compared with Commercial Sorbents......... 9

Bibliography for supporting information........ 10

1. Preparation and characterization of poly(4-vinylpyridine) brushes grafted to montmorillonite.

Preparation of GPC involves: acid activating of montmorillonite (aa-MMT), grafting ATPES (aa-MMT-ATPES), surface initiating with BIB (aa-MMT-ATPES-BIB) and finally, polymerizing of 4-VP by surface-initiated atom transfer radical polymerization (SI-ATRP). We validated the four stages of the synthesis by X-ray photoelectron spectroscopy (XPS), Fourier-transform infrared spectroscopy (FTIR), X-ray diffraction

(XRD), thermal gravimetric analysis (TGA), elemental analysis and zeta potential analysis.

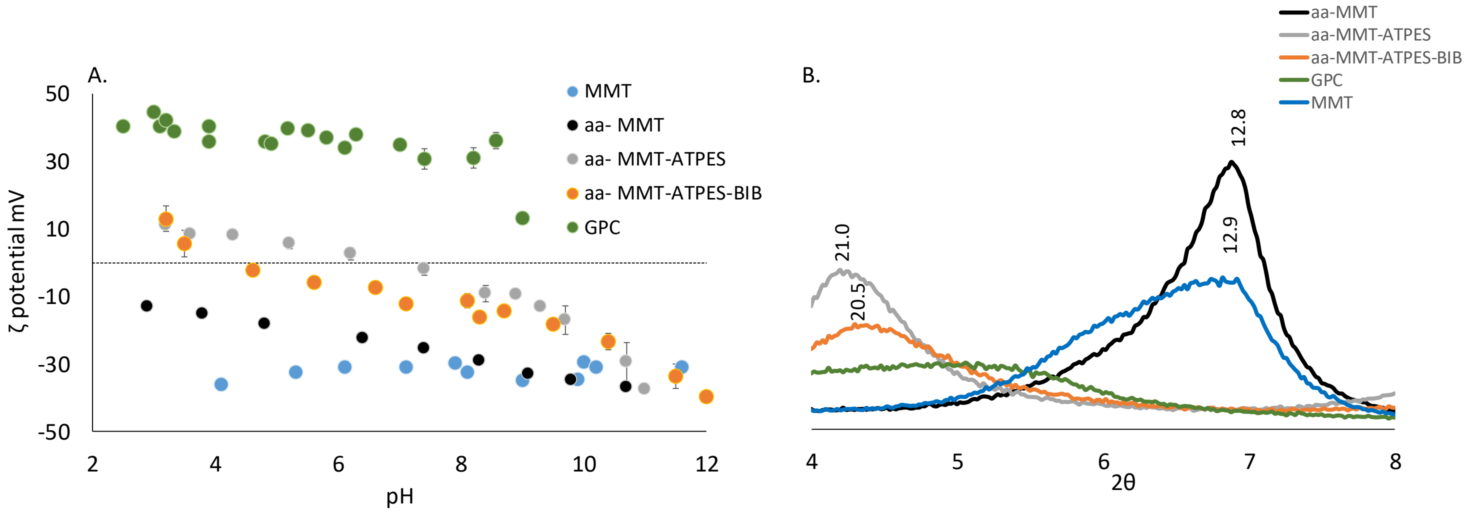

Figure S 1: A. presents the effect of suspension pH on the zeta potential of the different intermediate products of GPC synthesis. Zeta potential of both MMT and aa-MMT is negative at the pH range of 2-11. B. X-ray diffraction (XRD) patterns of oriented samples of intermediate products of GPC synthesis.

The zeta potential of MMT is constantly negative, reflecting the constant charge originating from the isomorphic substitutions in the mineral structure. The zeta potential of aa-MMT slightly increased with decreasing pH, indicating an increase in protonated sites (mainly Si-OH) which facilitated the grafting of APTES to the clay surface. The change in surface chemistry did not compromise the crystal structure of the clay, as the X-ray diffractions presented typical basal spacings (d=001) of 12.9 Å for both MMT and aa-MMT (Figure S 1B) [1].

ATPES was added to a suspension of aa-MMT in an ethanol/water mixture (75/25) [2] and as a result the zeta potential of aa-MMT-ATPES exhibited a high pH dependency, with charge reversal at pH~7.5 (Figure S 1A). At pH 10, the zeta potential of aa-MMT-

ATPES is -30 mV (like that of aa-MMT) because of deprotonation of the ATPES (pK_a=10.2). This indicated that the primary amine is not associated with the clay surface, but rather exposed to solution, supporting grafting to the clay surface via the silanol groups. The XRD of aa-MMT-ATPES revealed an increase in the clay basal spacing to 21 Å (compared with 12.9 Å for aa-MMT) due to the grafting of ATPES in between the clay platelets (Figure S 1). The mass percent of ATPES grafted to MMT reached 17%, as determined with TGA (Figure 4A). Shen et al. reported similar loadings and basal spacings for montmorillonite grafted with ATPES [1].

These results were also supported by FTIR measurements of aa-MMT-ATPES. A doublet peak (3311 and 3373 cm^{-1}), assigned to the primary amine, as well as peaks at 2850 and at 2930 cm^{-1} assigned to CH_3CH_2- stretching, appeared upon ATPES grafting (Figure S 2A). Elemental analysis of aa-MMT-ATPES confirmed a reduction in C/N molar ratio from 9 in $C_9H_{23}NO_3Si$ (ATPES) to 3.2 in aa-MMT-ATPES, which resulted from the condensation of three ethanol groups (theoretically C/N = 3) (Table S 1).

Binding BIB initiator to aa-MMT-ATPES changed the amine band in the FTIR spectra (Figure S 2B), in which the primary amine doublet disappears and only one peak, assigned to secondary amines, at 2933 cm^{-1} is present. Furthermore, the peak at 2976 cm^{-1}, assigned to CH_3 stretches, further supports the presence of the initiator on the surface. But, the zeta potentials of aa-MMT-ATPES-BIB are only slightly lower than aa-MMT-ATPES, suggesting that the BIB initiator did not fully modify the grafted ATPES (Figure S 1A). In fact, assuming full initiation should yield a C/N ratio of 6, based on Table S 1 we can roughly estimate that BIB attached to 57% of the ATPES.

Confirmation of vinylpyridine polymerization was obtained from the spectrum of aa-MMT-ATPES-BIB which was characterized by peaks at 1500 and at 1600-1640 cm^{-1}, corresponding to aromatic N=C and C=C, respectively (Figure S 2C). The loading of poly(4-vinylpyridine) brushes was 35% (w/w), determined from the substantial mass loss in the GPC thermogram at a temperature range of 200-500 ˚C (Figure S 3A). Similar values were reported for vinylpyridine SI-ATRP grafting on halloysite [3]. The theoretical C/N ratio of the polymer is 6, similar to the results in Table S1. Unreacted initiators and residual solvents can explain the slightly higher experimental values.

Furthermore, polymerization gave rise to clay exfoliation, as revealed from the X-ray diffractogram (Figure S 1B). In the case of poly(ethyl acrylate) brushes grafted to montmorillonite [4] the magnitude of d spacing increased as a function of the grafted amount with exfoliation obtained only upon SI-ATRP.

Table S 1: Element analysis after each of the four stages of synthesis.

Sample name	% N	% C	N (mmol/g)	C (mmol/g)	C/N (mol:mol)
aa-MMT	0.0	0.0	0.0	0.0	-
MMT-ATPES	2.7	7.4	2.0	6.2	3.2
aa-MMT-ATPES-BIB	3.6	14.8	2.6	12.4	4.8
GPC	5.6	31.8	4.0	26.5	6.6

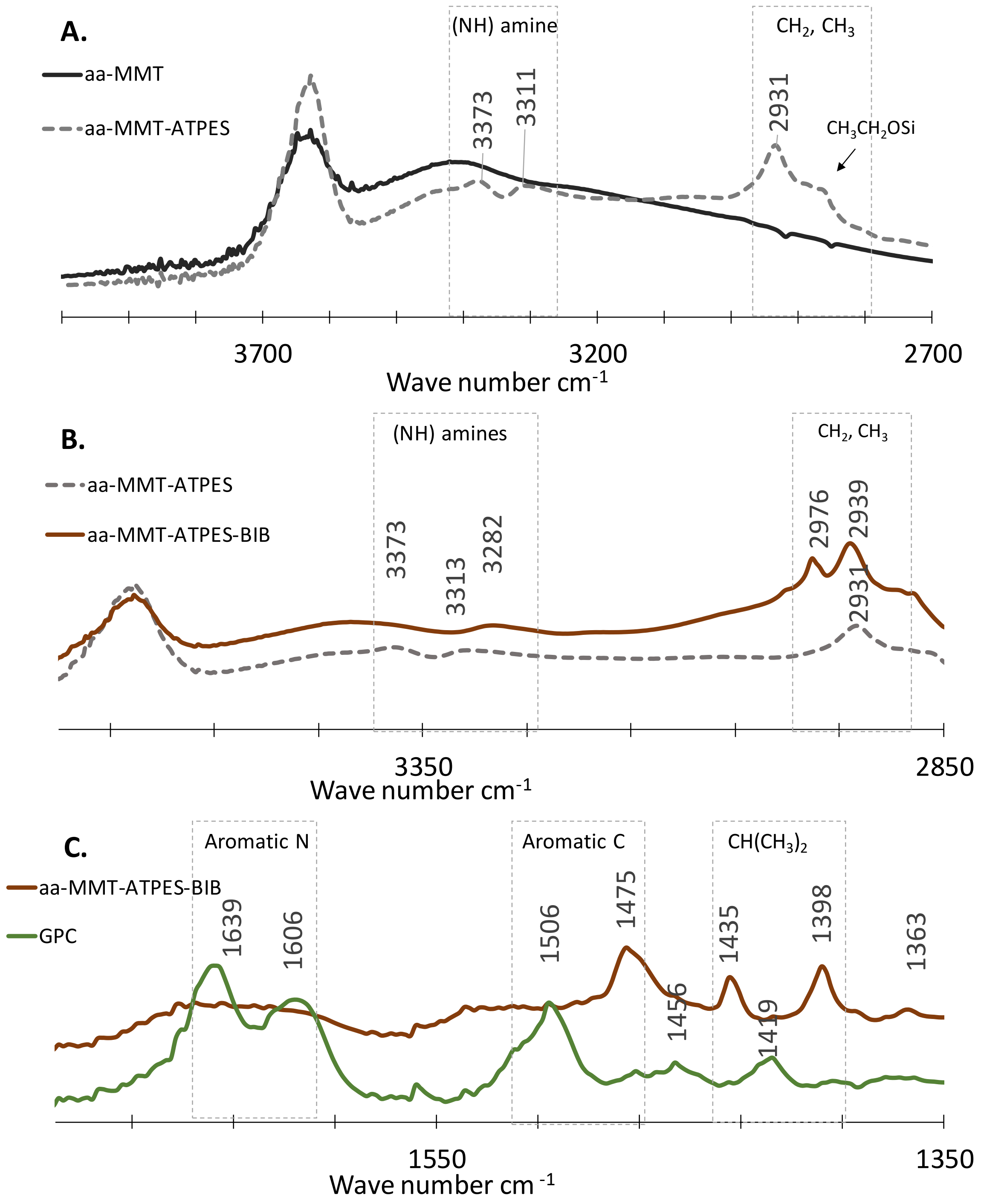

Figure S 1: FTIR-ATR spectra of the four stages of synthesis. A. aa-MMT and aa-MMT-ATPES, B. aa-MMT-ATPES and aa-MMT-ATPES-BIB. C. aa-MMT-ATPES-BIB and GPC.

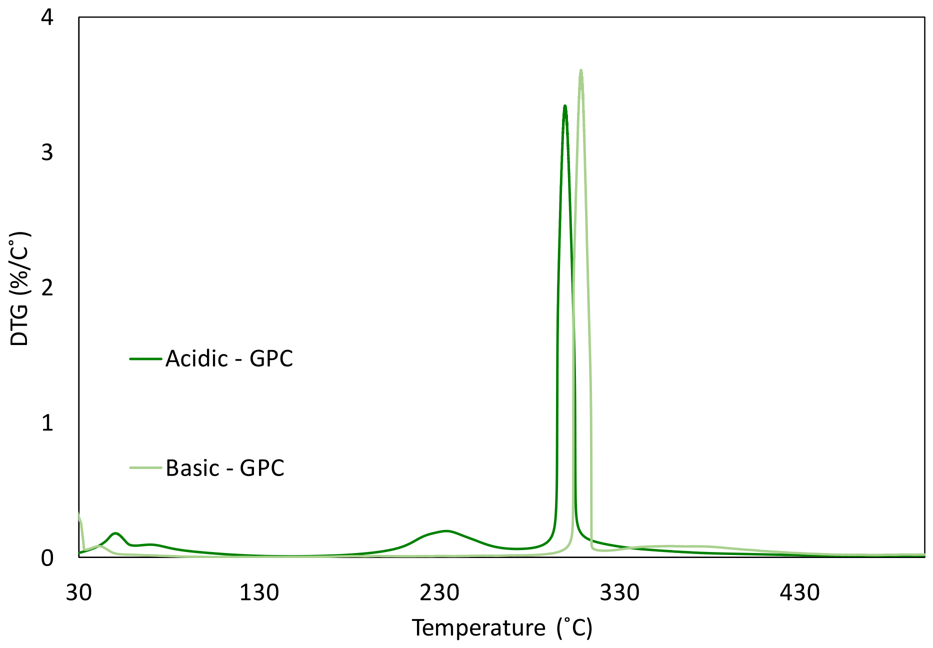

Figure S 3: Derivative thermal analysis (DTG) of GPC from an acidic pH and from a basic pH.

GPC Adsorption Performances in Comparison with Other Sorbents

Adsorption Capacity at Equilibrium

The adsorption capacity deduced from the Langmuir model (Q_m) in mmol per kg sorbent is presented in Table S 2. The capacity of GPC is significantly high or among the highest reports towards selenate and eosin-Y respectively. The removal of methyl blue (0.0025-0.2 mM) was almost complete by 2 g/L GPC, therefore fitting was not possible, and the capacity reported below is the measured amount.

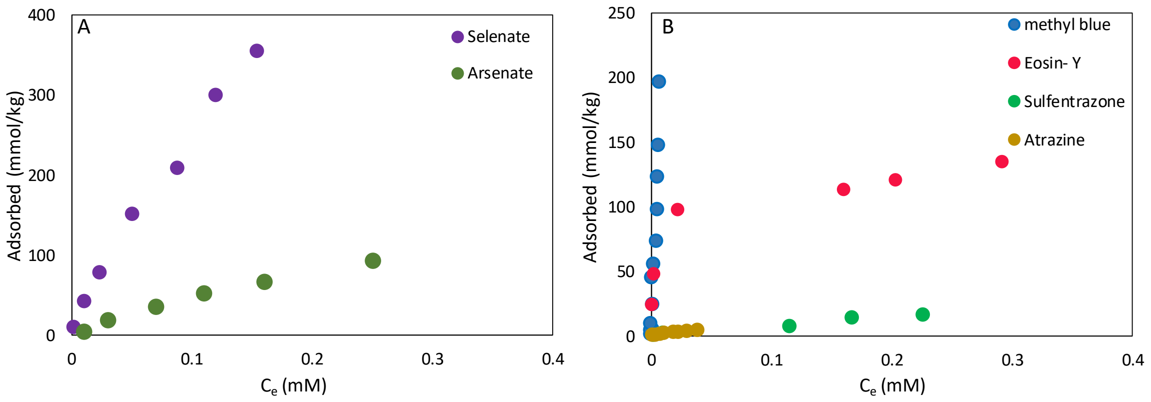

Figure S *4*: A. Adsorption isotherms (pH 3) of the inorganic oxyanions selenate and arsenate. B. Adsorption isotherms (pH 3) of the organic pollutants atrazine, sulfentrazone, eosin Y and methyl blue on 2 g L^{-1} GPC.

Table S 2: Comparison to the Langmuir adsorption capacity of reported sorbents for selenate, eosin Y and methyl blue.

Sorbent	Q_m (mmol kg^{-1})	R^2	Reference
	Selenate Removal		
Aluminum Oxide Coated sand (pH 4.9)	11.95	0.97	[5]
Chitosan–Clay Composite	233.03	0.97	
Fe-oxide	103.85	0.97	[6]
Al-oxide	217.83	0.97	
iron-coated GAC	32.67	0.99	[7]
Iron Oxide	107.27	0.99	[8]
Silicon Oxide	89.41	0.99	

Functionalized graphene Oxide	771.27	0.98	[9]
Zirconium-based MOF	1076.5	0.97	[10]
modified magnetic graphene Oxide	1490	0.88	[11]
conjugate adsorbent	1184	0.99	[12]
MgO nanosheets	1310	0.99	[13]
chitosan based nanocomposite	197.88	0.99	[14]
hematite modified magnetic	316.6	0.99	[15]
GPC	1232	0.99	This study
Eosin Y removal			
Anaerobic Sludge	28.02	0.98	[16]
Chitosan Hydrobeads	116.76	~0.99	[17]
MWCNT's	158.78	0.99	[18]
TEPA Modified Sugarcane Bagasse	576.64	0.98	[19]
PEDEA Modified Bentonite	127.74	0.99	[20]
Nickel Nanoparticles	0.88	0.98	[21]
N-doped Zinc Oxide	1.85	0.83	[22]
Polyaniline	620	0.99	[23]
polydopamine microspheres	<14	NA	[24]

Sol-Gel γ-Al2O3	69.09	0.99	[25]
GPC	123.3	0.94	This study
	Methyl Blue removal		
Magnetic Chitosan Grafted Graphene	142.39	0.99	[26]
strontium and barium phosphate nanorods	2114	0.99	[27]
GPC [a]	>196.29	n.a	This study

[a] Measured capacity

Filtration Performances, Compared with Commercial Sorbents.

The removal of selenate and eosin-Y by sorbent-filled filtration columns is described below in Table S 3. For the removal of selenate, a commercial ion exchange resin recommended for oxyanions removal was selected. Activation of the resin column was performed by flowing the manufacturer instructions and the activation of GPC columns is described in the materials and methods section. The removal of eosin-Y by GPC columns was compared to granular activated carbon columns as it is the commonly used sorbent for adsorption in filtration columns. The removal of selenate by GPC was higher than by the commercial resin while the removal of eosin-Y was significantly higher by GPC than by GAC (Table S 3).

Table S 3: Column filtration of selenate by GPC and by PWA resin and eosin-Y by GPC and granular activated carbon (GAC) in the first filtration cycle.

Selenate Removal	
Filtrated volume mL	Se Adsorbed %

GPC	PWA Resin	GPC	PWA Resin
86	93	100 ± 0	91 ± 1
157	170	100 ± 0	90 ± 0
229	247	100 ± 0	91 ± 1
415	448	100 ± 0	91 ± 0
515	556	100 ± 0	91 ± 1

Eosin-Y Removal			
Filtrated volume mL		Se Adsorbed %	
GPC	GAC	GPC	GAC
33	26	100 ± 0	7 ± 0
59	61	100 ± 0	4 ± 1
138	138	100 ± 0	4 ± 1
170	182	100 ± 0	5 ± 1
275	338	30 ± 0	4 ± 1

Bibliography for supporting information

[1] Shen W, He H P, Zhu J X, Yuan P, Ma Y H and Liang X L 2009 Preparation and characterization of 3-aminopropyltriethoxysilane grafted montmorillonite and acid-activated montmorillonite *Chinese Science Bulletin* **54** 265–71

[2] He H, Duchet J, Galy J and Gerard J F 2005 Grafting of swelling clay materials with 3-aminopropyltriethoxysilane *Journal of Colloid and Interface Science* **288** 171–6

[3] Jiang J, Zhang Y, Cao D and Jiang P 2013 Controlled immobilization of methyltrioxorhenium(VII) based on SI-ATRP of 4-vinyl pyridine from halloysite nanotubes for epoxidation of soybean oil *Chemical Engineering Journal* **215–216** 222–6

[4] Matsumura S, Hlil A R, Lepiller C, Gaudet J, Guay D, Shi Z, Holdcroft S and Hay A S 2008 Ionomers for proton exchange membrane fuel cells with sulfonic acid

groups on the end-groups: Novel branched poly(ether-ketone)s *American Chemical Society, Polymer Preprints, Division of Polymer Chemistry* **49** 511–2

[5] Kuan W-H, Lo S-L, Wang M K and Lin C-F 1998 Removal of Se(IV) and Se(VI) from water by aluminum-oxide-coated sand *Water Research* **32** 915–23

[6] Bleiman N and Mishael Y G 2010 Selenium removal from drinking water by adsorption to chitosan-clay composites and oxides: batch and columns tests. *Journal of Hazardous Materials* **183** 590–5

[7] Zhang N, Lin L S and Gang D 2008 Adsorptive selenite removal from water using iron-coated GAC adsorbents *Water Research* **42** 3809–16

[8] Sheha R R and El-Shazly E A 2010 Kinetics and equilibrium modeling of Se(IV) removal from aqueous solutions using metal oxides *Chemical Engineering Journal* **160** 63–71

[9] Xiao W, Yan B, Zeng H and Liu Q 2016 Dendrimer functionalized graphene oxide for selenium removal *Carbon* **105** 655–64

[10] Howarth A J, Katz M J, Wang T C, Platero-Prats A E, Chapman K W, Hupp J T and Farha O K 2015 High Efficiency Adsorption and Removal of Selenate and Selenite from Water Using Metal-Organic Frameworks *Journal of the American Chemical Society* **137** 7488–94

[11] Lu Z, Yu J, Zeng H and Liu Q 2017 Polyamine-modified magnetic graphene oxide nanocomposite for enhanced selenium removal *Separation and Purification Technology* **183** 249–57

[12] Awual M R, Hasan M M and Khaleque M A 2015 Efficient selenium(IV) detection and removal from water by tailor-made novel conjugate adsorbent *Sensors and Actuators, B: Chemical* **209** 194–202

[13] Cui W, Li P, Wang Z, Zheng S and Zhang Y 2018 Adsorption study of selenium ions from aqueous solutions using MgO nanosheets synthesized by ultrasonic method *Journal of Hazardous Materials* **341** 268–76

[14] Seyed Dorraji M S, Amani-Ghadim A R, Hanifehpour Y, Woo Joo S, Figoli A,

Carraro M and Tasselli F 2017 Performance of chitosan based nanocomposite hollow fibers in the removal of selenium(IV) from water *Chemical Engineering Research and Design* **117** 309–17

[15] Ma Z, Shan C, Liang J and Tong M 2018 Efficient adsorption of Selenium(IV) from water by hematite modified magnetic nanoparticles *Chemosphere* **193** 134–41

[16] Wang Y, Mu Y, Zhao Q B and Yu H Q 2006 Isotherms, kinetics and thermodynamics of dye biosorption by anaerobic sludge *Separation and Purification Technology* **50** 1–7

[17] Chatterjee S, Chatterjee S, Chatterjee B P, Das A R and Guha A K 2005 Adsorption of a model anionic dye, eosin Y, from aqueous solution by chitosan hydrobeads *Journal of Colloid and Interface Science* **288** 30–5

[18] Yu H and Fugetsu B 2010 A novel adsorbent obtained by inserting carbon nanotubes into cavities of diatomite and applications for organic dye elimination from contaminated water *Journal of Hazardous Materials* **177** 138–45

[19] Jiang G B, Lin Z T, Huang X Y, Zheng Y Q, Ren C C, Huang C K and Huang Z J 2012 Potential biosorbent based on sugarcane bagasse modified with tetraethylenepentamine for removal of eosin Y *International Journal of Biological Macromolecules* **50** 707–12

[20] Kang Q, Zhou W, Li Q, Gao B, Fan J and Shen D 2009 Adsorption of anionic dyes on poly(epicholorohydrin dimethylamine) modified bentonite in single and mixed dye solutions *Applied Clay Science* **45** 280–7

[21] Pandian C J, Palanivel R and Dhananasekaran S 2015 Green synthesis of nickel nanoparticles using Ocimum sanctum and their application in dye and pollutant adsorption *Chinese Journal of Chemical Engineering* **23** 1307–15

[22] Ramachandran S and Sivasamy A 2016 Synthesis and characterization of nanocrystalline N-doped semiconductor metal oxide and its visible photocatalytic activity in the degradation of an organic dye *Journal of Environmental Chemical Engineering*

[23] Bahramifar N, Tavasolli M and Younesi H 2015 Removal of eosin Y and eosin B dyes from polluted water through biosorption using Saccharomyces cerevisiae : Isotherm , kinetic and thermodynamic studies *Journal of Applied Research in Water and Wastewater* **3** 108–14

[24] Fu J, Xin Q, Wu X, Chen Z, Yan Y, Liu S, Wang M and Xu Q 2016 Selective adsorption and separation of organic dyes from aqueous solution on polydopamine microspheres *Journal of Colloid and Interface Science* **461** 292–304

[25] Thabet M S and Ismaiel A M 2016 Sol-Gel γ-Al2O3 Nanoparticles Assessment of the Removal of Eosin Yellow Using : Adsorption , Kinetic and Thermodynamic Parameters *Journal of Encapsulation and Adsorption Sciences* **6** 70–90

[26] Fan L, Luo C, Li X, Lu F, Qiu H and Sun M 2012 Fabrication of novel magnetic chitosan grafted with graphene oxide to enhance adsorption properties for methyl blue *Journal of Hazardous Materials* **215–216** 272–9

[27] Zhang F, Song W and Lan J 2015 Effective removal of methyl blue by fine-structured strontium and barium phosphate nanorods *Applied Surface Science* **326** 195–203
